# Mucoepidermoid Carcinoma of the Intrapancreatic Common Bile Duct: Immunohistochemical Profile, Prognosis, and Review of the Literature

**DOI:** 10.1155/2013/192458

**Published:** 2013-12-03

**Authors:** Adrienne E. Moul, Pablo A. Bejarano, Javier Casillas, Joe U. Levi, Monica T. Garcia-Buitrago

**Affiliations:** ^1^Department of Pathology, Jackson Memorial Hospital, University of Miami, Miami, FL 33136, USA; ^2^Department of Pathology, Cleveland Clinic Florida, Weston, FL 33331, USA; ^3^Department of Radiology, Jackson Memorial Hospital, University of Miami, Miami, FL 33136, USA; ^4^Department of Surgery, Jackson Memorial Hospital, University of Miami, Miami, FL 33136, USA; ^5^University of Miami Miller School of Medicine, 1611 NW 12th Avenue, Holtz Center, Room 2042 E, Miami, FL 33136, USA

## Abstract

Mucoepidermoid carcinoma of the bile duct is a rare entity. Only one mucoepidermoid carcinoma from the common bile duct has been reported in the Korean literature. Herein, we present the first in the English literature. The tumor arose in the intrapancreatic (distal) common bile duct in an 83-year-old woman who presented with obstructive jaundice and elevated liver enzymes. The tumor invaded the underlying pancreas and peripancreatic adipose tissue and showed pagetoid spread into the extrapancreatic common bile duct and cystic duct. The tumor exhibited nests of malignant cells with diffuse CK7 and MUC1 positivity. The basal cells were p63 and CK5/6 positive. The luminal cells were stained with carcinoembryonic antigen, MUC5, and mucicarmine and were focally positive for CK20. There was focal MUC4 staining on the apical luminal border. The neoplastic cells were negative for MUC2 and HER2-neu. We discuss the clinical presentation, diagnostic features, immunohistochemical profile, and prognosis of mucoepidermoid carcinoma of the common bile duct. The features of this neoplasm are further compared with mucoepidermoid carcinoma of the hepatobiliary system, adenosquamous carcinoma, and mucoepidermoid carcinoma of other organs.

## 1. Introduction

Mucoepidermoid carcinoma (MEC) occurs in various organs including the salivary glands, lung, and pancreas [1–3]. Its presence in the biliary system is rare [4–16]. Only one case of mucoepidermoid carcinoma arising from the distal common bile duct has been reported in the Korean literature [10]. We present the first reported case of mucoepidermoid carcinoma of the common bile duct in the English literature and discuss the clinical presentation, diagnostic features, immunohistochemical profile, its prognosis when compared to mucoepidermoid carcinoma from other sites, and its most common differential, adenosquamous carcinoma.

## 2. Case Presentation

**Figure 1:**
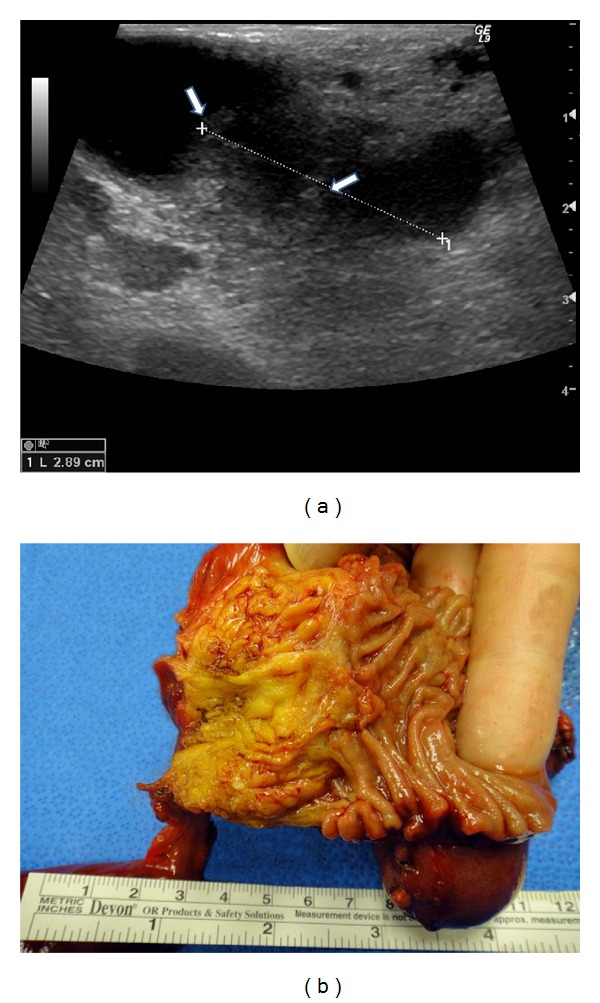
(a) Intraoperative ultrasound demonstrating a dilated common bile duct, diameter 1.3 cm. Note a hypoechoic amorphous mass in the distal lumen of this duct (arrows). (b) Gross picture of the ampullary region showing a 2.0 cm indurated area of the intrapancreatic (distal) common bile duct.

An 83-year-old female was referred to our institution after being evaluated at an outside institution for obstructive jaundice with elevated liver function tests, where computed tomography (CT) imaging and magnetic resonance cholangiopancreatography (MRCP) revealed dilatation of the intra- and extrahepatic biliary system with an abrupt cutoff of the distal common bile duct. No congenital cysts were seen. The patient underwent a transhepatic cholangiogram and a transhepatic cholangiocatheter was placed for decompression. There was initial improvement to the patient's symptoms. However, the direct bilirubin continued to be elevated at 6.68 mg/dL and the catheter later became dislodged. Endoscopic retrograde cholangiopancreatogram (ERCP) was attempted, but it was unsuccessful. Preoperative laboratory tests demonstrated elevated cancer antigen 19-9 of 980.2 U/mL (normal 0–30.9 U/mL), elevated alkaline phosphatase of 940 U/L (normal 20–130 U/L), and hyperbilirubinemia of 13.1 mg/dL (normal 0.1–1.1 mg/dL). The patient underwent an exploratory laparotomy. Intraoperative ultrasound showed a dilated common bile duct (1.3 cm in diameter) with distal obstruction due to a 2.0 cm periductal hypoechoic mass (Figure 1(a)). A pylorus-sparing pancreaticoduodenectomy (Whipple) procedure was performed. During the following seven postoperative months, the patient received 4 cycles of chemotherapy with gemcitabine (800 mg/m^2^), abdominal radiotherapy, and 5-fluorouracil (1575 mg/m^2^) as radiosensitizer. Four months after completing adjuvant therapy, the patient was found to have multiple ring-enhancing liver lesions in both lobes, measuring up to 2 cm. Two months later, the patient expired.

The entire tumor was fixed in formalin and embedded in paraffin. Four-micron sections were cut and stained with hematoxylin and eosin. Tumor sections were stained with mucicarmine stain (Dako) and immunohistochemistry using the Dako EnVision system with the following antibodies: CK7 (Dako, mouse monoclonal, RTU), CK20 (Dako, mouse monoclonal, RTU), carcinoembryonic antigen (CEA, Dako, mouse monoclonal, RTU), p63 (BioCARE, mouse monoclonal, 1 : 20), CK5/6 (Dako, mouse monoclonal, RTU), MUC1 (BioCARE, mouse monoclonal, 1 : 100), MUC2 (Dako, mouse monoclonal, RTU), MUC4 (Invitrogen, mouse monoclonal, 1 : 400), MUC5 (Leica, mouse monoclonal, 1 : 50), and HER2-neu (c-erbB2, Dako, rabbit polyclonal, 1 : 3000).

Fluorescence in situ hybridization (FISH) was performed on formalin fixed paraffin embedded tissue using ZytoVision LSI mastermind-like 2 (MAML2) (11q21) dual-color break-apart probe according to the manufacturer's protocol.

Examination of the Whipple specimen with the attached gallbladder revealed an indurated area around the intrapancreatic (distal) common bile duct, measuring 2 × 1.5 × 0.5 cm (Figure 1(b)). The tumor grossly invaded the underlying pancreas and peripancreatic adipose tissue but did not invade the duodenum. The ampulla was grossly unremarkable.

**Figure 2:**
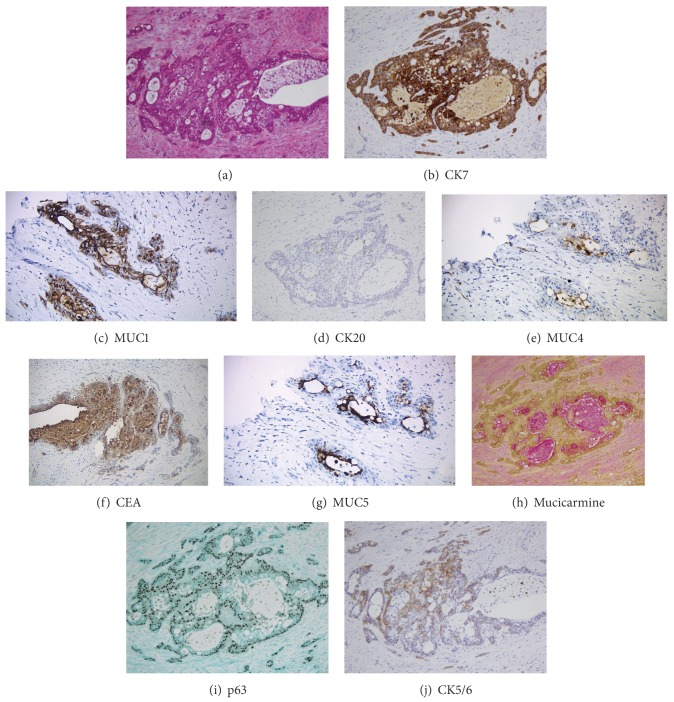
(a) Nest of mucin-secreting tumor cells, lined by squamoid tumor cells (H&E, 20x). (b) Diffuse CK7 positivity (CK7 stain, 20x). (c) Tumor cells immunoreactive for MUC1 (MUC1 stain, 20x). (d) Glandular luminal mucin-secreting tumor cells focally stained for CK20 (CK20 stain, 20x). (e) MUC4 positivity in the apical aspect of luminal mucin-secreting tumor cells (MUC4 stain, 20x). (f) CEA positivity in glandular luminal tumor cells (CEA stain, 20x). (g) MUC5 staining in the luminal tumor cells (MUC5 stain, 20x). (h) Mucicarmine stain highlighting the intracytoplasmic mucin in the tumor cells (mucicarmine stain, 20x). (i) Outer squamoid tumor cells showed diffuse p63 staining (p63 stain, 20x). (j) Outer squamoid tumor cells showed diffuse CK5/6 staining (CK5/6 stain, 20x).

The tumor demonstrated ductal-like nests composed of epithelial squamoid and mucous cells. Intermediate cells were sparse (Figure 2(a)). The tumor focally invaded the underlying pancreas and peripancreatic adipose tissue. In addition, it showed pagetoid spreading into the extrapancreatic common bile duct and cystic duct but did not invade the gallbladder. Surgical resection margins were negative. There was direct extension of carcinoma to one out of 14 lymph nodes and extensive perineural invasion. The pathologic staging was pT3 N1 M n/a, based on the AJCC classification 7th edition [17].

The tumor cells were diffusely positive for CK7 and MUC1 and negative for MUC2 and HER2-neu. The glandular luminal tumor cells were positive for MUC5 and CEA and focally positive for CK20 and MUC4 (apical luminal) (Figures 2(b)–2(g)). The basal squamoid cells within the tumor nests were positive for p63 and CK5/6 (Figures 2(i) and 2(j)). Mucicarmine stain highlighted the intracytoplasmic mucin present within glandular luminal cells in these nests (Figure 2(h)). The histomorphology and immunohistochemical profile are diagnostic for mucoepidermoid carcinoma.

Interphase fluorescence in situ hybridization (FISH) analysis for the mastermind-like 2 gene (MAML2) on chromosome 11q22 was negative.

## 3. Discussion

Mucoepidermoid carcinoma of the intra- and extrahepatic bile ducts is extremely rare. Koo et al. described the only two cases that have been reported in the perihilar common hepatic bile duct, and only eighteen cases have been reported in the intrahepatic bile ducts [4–9, 11–16]. Song et al. of Korea (Table 1) reported the first mucoepidermoid carcinoma of the intrapancreatic common bile duct in 2011 [10].

Koo et al. suggested that MEC may be related to *Clonorchis sinensis* infestation with or without chronic bacterial infection because three of the five reported patients at that time showed evidence of clonorchiasis and two patients had primary recurrent pyogenic cholangitis [5]. It has been also reported that MEC may arise from preexisting congenital cysts [6, 7] in which the epithelium may transform into pluripotent intermediate cells, capable of differentiating into both mucin-secreting and squamous cells [6]. Similarly, Onoda et al. demonstrated by electron microscopy that undifferentiated cells of the pancreatic duct showed multipotency [3]. Other possible etiologic associations included thorotrast contrast [8] and intrahepatic stones [6].

The diagnosis of MEC has been established using mucicarmine and Alcian blue and/or Periodic Acid Schiff (PAS) stains to demonstrate the presence of mucin within the tumor [4–8, 11–16]. A few authors used electron microscopy to demonstrate the presence of tonofibrils or desmosomes in the squamoid component and mucin granules and microvilli in the mucus-producing cells [6, 8, 13, 14]. Recent literature of mucoepidermoid carcinomas of the intrahepatic bile duct has reported CK7 positivity, one also having diffuse CK20 positivity [4, 6]. The mucoepidermoid carcinoma of the intrapancreatic bile duct described by Song et al. showed p16 positivity [10]. In 2005, Handra-Luca et al. examined the expression of MUC proteins in salivary gland MECs in relation to their diagnostic and prognostic implications. MUC1 and MUC4 stained the apical portion of glandular tumor cells and the membrane of intermediate and epidermoid tumor cells while MUC2 and MUC5AC stained the cytoplasm of glandular, mucous, and intermediate tumor cells. MUC1 expression correlated with shorter progression-free survival. Complete lack of MUC4 was associated with poor differentiation, neural invasion, necrosis, and anaplasia [18]. Our case demonstrated diffuse CK7 and MUC1 positivity staining in both the glandular and basal squamoid cells. The glandular luminal cells were positive for CEA, MUC5, and mucicarmine and showed focal apical CK20 and MUC4 positivity. The basal cells were positive for CK5/6 and p63. Squamoid cells predominated, whereas intermediate cells were sparse. While a few authors specifically mention scarce intermediate cells [4, 11], this is unlike the MEC of the salivary glands, which have a predominance of intermediate cell population [1].

Prognosis of mucoepidermoid carcinoma of the hepatobiliary system is poor. Arakawa et al. reported that one year after diagnosis of intrahepatic bile duct carcinoma only one out of seventeen patients was alive and one patient was lost to followup [4]. Our patient was found to have metastasis to the liver in her eleventh postoperative month and expired thirteen months after surgery. Song et al. reported that their patient was on supportive treatment after developing liver metastasis twelve months after initial treatment (Table 1) [10]. Koo et al. described two patients with tumor in the common hepatic bile duct; one expired 6 months after surgery and one was alive ten months after surgery when the cases were reported [5].

Unlike those with mucoepidermoid carcinoma of the hepatobiliary system, most patients diagnosed with mucoepidermoid carcinoma of the salivary glands have a favorable outcome. The high-grade and MUC1-positive MECs have a worse prognosis [1, 18].

The chromosomal translocation of mastermind-like gene family (MAML2) located on chromosome 11q21 with the N-terminal cAMP response element-binding protein (CREB) binding domain of CREB-regulated transcription coactivator 1 (CRTC1) at 19q21 has been shown to be a highly specific translocation in mucoepidermoid carcinomas of the salivary gland [19]. In the literature, the translocation has been found anywhere from 40 to 80% of all MECs, with the low and intermediate grades having a higher percentage of translocations [19, 20]. Studies have suggested that this translocation is considered an excellent prognosis in high-risk patients [20]. There are no reports of this translocation in MEC of the hepatobiliary system. In our case, no MAML2 translocation was detected. However, our tumor is a high-grade lesion, and the high-grade MEC of the salivary glands has a lower percentage of the translocation. The main differential for mucoepidermoid carcinoma is adenosquamous carcinoma in various organs. The World Health Organization defines mucoepidermoid carcinoma as a tumor characterized by a combination of mucin-secreting, squamous, and intermediate cells [1], different from adenosquamous carcinoma [21], which is a biphasic tumor consisting of two different components. The adenocarcinoma component contains ductal or glandular structures with focal to abundant intracellular or extracellular mucin while the squamous component is characterized by infiltrating nests or sheets of polygonal cells with distinct cellular borders, intercellular bridges, opaque eosinophilic cytoplasm, and varying degrees of keratinization. The two different components can be seen separated topographically within the substance of the tumor or intimately amalgamated with one another. Our case did not show separate components or keratinization. Adenosquamous carcinoma is a rare subtype of extrahepatic bile duct (EBD) carcinoma, with an incidence of 2–5% [22]. In a study by Hong et al., it was concluded that patients with EBD adenosquamous carcinoma had a significantly worse prognosis than those with EBD adenocarcinoma (11-month verses 32-month median survival). Moreover, adenosquamous carcinoma patients with a squamous cell carcinoma component at the advancing edge had even worse survival time when compared to those with an adenocarcinoma component (median survival 6 months versus 29 months) [22].

Due to the limited reported cases of mucoepidermoid carcinoma in the hepatic bile duct and intrapancreatic common bile duct, a direct comparison to adenosquamous carcinoma regarding prognosis is difficult to make. However, since MECs do not contain pure adenocarcinoma areas to favorably prognosticate these tumors, we hypothesize that MECs have even worse prognosis than adenosquamous carcinoma. Therefore, classical MECs of the bile ducts should be subcharacterized as a morphological variant different from adenosquamous carcinoma, like in other organs. More case series will be needed to determine the clinicopathological significance for distinguishing mucoepidermoid carcinomas and adenosquamous carcinomas in the hepatobiliary system.

In conclusion, we present the second reported case of mucoepidermoid carcinoma of the intrapancreatic (distal) common bile duct, which showed aggressive biological behavior as the previously reported MEC of the intra- and extrahepatic bile ducts. Our case emphasizes the use of immunohistochemistry to characterize MEC and differentiate it from adenocarcinoma and adenosquamous carcinoma. Awareness of this morphological variant may have important prognostic implications, which in the future may help to select and improve the patient selection for adjuvant therapies and overall survival.

## Figures and Tables

**Table 1:** Reported cases of mucoepidermoid carcinoma of the distal common bile duct [10].

Author	Age, sex	Location	Size	Expansion	Treatment	Followup
Song et al. (2011) [10]	68, M	Intrapancreatic common bile duct	4.7 cm	Lymph node metastasis (2/17)	5-FU and radiation total dose 500 cGY)	Developed metastasis to liver. Patient on supportive care 12 months after initial treatment, when published.
Moul et al. (current case) (2013)	83, F	Intrapancreatic common bile duct, cystic duct	2 cm	Infiltrated pancreas and peripancreatic soft tissue, lymph node metastasis (1/14)	4 cycles of gemcitabine, 5-FU, and radiation	Developed metastasis to the liver. Expired 13 months after surgery.
